# CS-AMPPred: An Updated SVM Model for Antimicrobial Activity Prediction in Cysteine-Stabilized Peptides

**DOI:** 10.1371/journal.pone.0051444

**Published:** 2012-12-11

**Authors:** William F. Porto, Állan S. Pires, Octavio L. Franco

**Affiliations:** Centro de Análises Proteômicas e Bioquímicas, Pós-Graduação em Ciências Genômicas e Biotecnologia Universidade Católica de Brasília, Brasília-DF, Brazil; University of South Florida College of Medicine, United States of America

## Abstract

The antimicrobial peptides (AMP) have been proposed as an alternative to control resistant pathogens. However, due to multifunctional properties of several AMP classes, until now there has been no way to perform efficient AMP identification, except through *in vitro* and *in vivo* tests. Nevertheless, an indication of activity can be provided by prediction methods. In order to contribute to the AMP prediction field, the CS-AMPPred (Cysteine-Stabilized Antimicrobial Peptides Predictor) is presented here, consisting of an updated version of the Support Vector Machine (SVM) model for antimicrobial activity prediction in cysteine-stabilized peptides. The CS-AMPPred is based on five sequence descriptors: indexes of (i) α-helix and (ii) loop formation; and averages of (iii) net charge, (iv) hydrophobicity and (v) flexibility. CS-AMPPred was based on 310 cysteine-stabilized AMPs and 310 sequences extracted from PDB. The polynomial kernel achieves the best accuracy on 5-fold cross validation (85.81%), while the radial and linear kernels achieve 84.19%. Testing in a blind data set, the polynomial and radial kernels achieve an accuracy of 90.00%, while the linear model achieves 89.33%. The three models reach higher accuracies than previously described methods. A standalone version of CS-AMPPred is available for download at <http://sourceforge.net/projects/csamppred/> and runs on any Linux machine.

## Introduction

Microorganisms may cause enormous problems in diverse fields, including human health and agribusiness. In the last few decades, many microorganisms have developed resistance against a number of antimicrobial agents. In this context, the antimicrobial peptides (AMP) have been proposed as an alternative to control such dangerous microorganisms [1]. The AMPs can perform different functions under different environmental conditions. This ability is also known as 'peptide promiscuity' [2]. According to Franco (2011) [2], there are two levels of multifunctionality, where on the first level, a single peptide can perform diverse functions; and on the second level, a peptide superfamily has members with different functions and/or members with multiple activities, which could be related to different exposed residues in the same structural framework [2].

These compounds have been isolated from several sources, in all life kingdoms [1], [3], and they can be classified in two major groups, according to the presence or absence of disulphide bridges [3]. The disulphide-free peptides are composed mainly of α-helical and unstructured AMPs; while the cysteine-stabilized AMPs are composed of several classes, which are divided according to their disulphide patterns. The cysteine-stabilized peptides can be related to both multifunctional behaviors [2], [4], with a strong tendency to have superfamily multifunctionality.

Family’s multifunctional behavior has been linked to special events, such as gene duplication, which allow the generation of novel protein functions derived from the ability of a protein to adopt a new function based on the modification of a few amino acid residues in an existing fold [2], [5]. Those modifications can have effects, slight or not, on the pivotal function, being able to yield a totally unusual function. Therefore, the structure-activity relationship is controversial for AMPs, since this relationship is becoming more and more unclear [2].

This kind of behavior can be observed in several cysteine-stabilized peptides, including the ones which are restricted to one life kingdom, such as the α defensins from vertebrates [6], [7]; the cyclotides [8], [9] and the thionins [10], [11] from plants; and also observed in classes which can be found in more than one life kingdom, such as the CSαβ defensins, which can be found in plants [12], [13], insects [14], [15] and fungi [16], [17], [18]; and the hevein-like peptides, which can be found in plants and fungi [4], [19].

Recently, it has been proposed that physicochemical properties can be used as descriptors to predict the antimicrobial activity of cysteine-stabilized peptides by means of machine learning methods [20]. Several studies have applied machine learning methods for antimicrobial activity prediction [20]–[26]. These methods aim to identify AMPs prior to *in vitro* tests, so that antimicrobial sequences can be identified directly from protein databases and further expressed in heterologous systems or synthesized [21], [26].

In protein data bases, several sequences are annotated as hypothetical, unnamed or unknown proteins, including sequences that resemble antimicrobial peptides [4], [27]. An easy way to explore the protein databases consists of searching for sequences through patterns or another similarity search approach, such as local alignments [17]. This kind of approach is commonly applied to cysteine-stabilized antimicrobial peptides, since the classes have a typical cysteine pattern. Indeed, the majority of plant AMPs are cysteine rich [27], [28], with only few examples of plant disulphide-free AMPs [29]–[33]. If compared to the peptide purification process, the database search has the advantages of fast sequence identification and low costs. Therefore, this kind of approach can be applied in a more general manner, searching for any small cysteine-rich peptides in plant genomes [27] or in a more specific manner, by searching for a specific AMP class against the whole database [4], [34].

However, since cysteine-stabilized AMPs are mostly multifunctional peptides, how is it possible to identify the sequences with antimicrobial activity? The answer will in fact be obtained only through *in vitro* and/or *in vivo* tests; however, the prediction methods can provide an indication of activity, improving the search methods. Bearing this in mind, here the CS-AMPPred (Cysteine-Stabilized Antimicrobial Peptides Predictor) is presented, as an updated version of the support vector machine (SVM) model proposed by our group [20] for antimicrobial activity prediction in cysteine-stabilized peptides.

**Figure 1:**
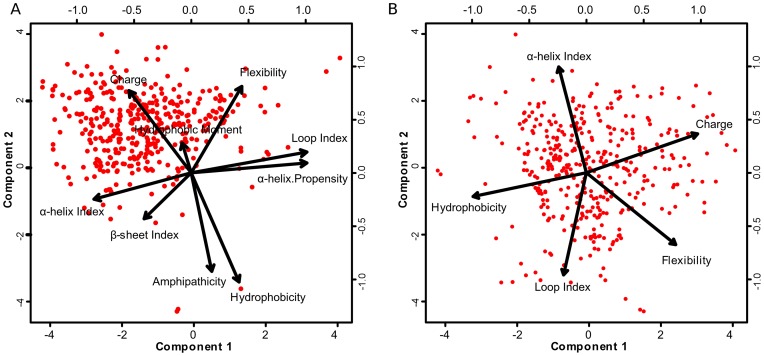
Principal component analysis of sequence descriptors for cysteine-stabilized peptides. The components are indicated by arrows: as larger the arrow is, major is the component contribution to the set’s variance. (A) The disposition of the nine sequence descriptors in the peptide space; (B) the final ensemble of descriptors, the descriptors hydrophobic moment, index of β-sheet formation, rate between charged and hydrophobic residues and α-helix propensity were ruled out.

**Figure 2:**
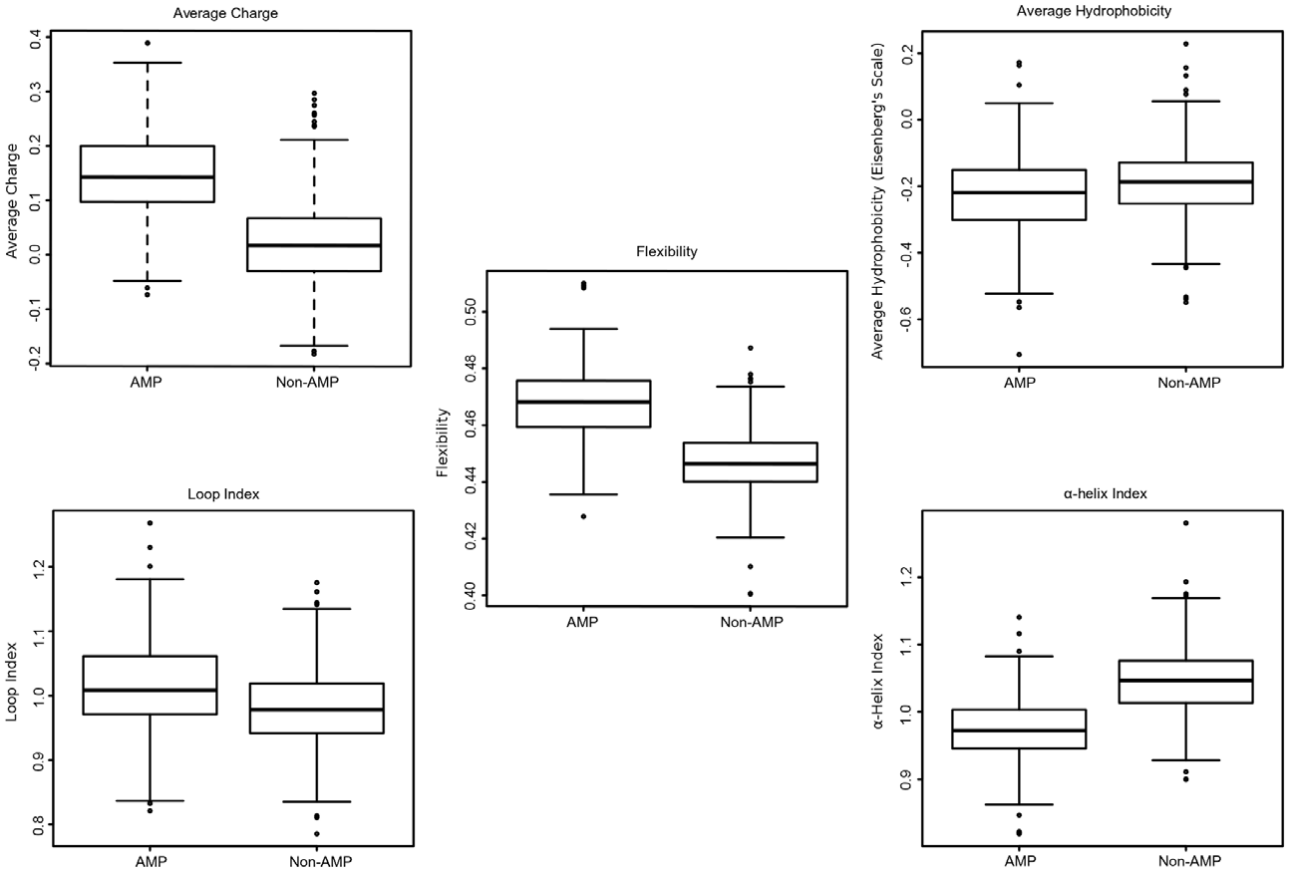
Distribution of sequence descriptor values. The left box in each panel corresponds to the AMPs. All descriptors have statistical differences when compared to the non-antimicrobial data set, with a critical value of 0.05. The observed p-values are as follows: charge (<2.2e-16), hydrophobicity (2.169e-06), flexibility (<2.2e-16), index of α-helix formation (<2.2e-16) and index of loop formation (2.908e-10).

## Materials and Methods

### Data Sets

The positive data set (PS) was constructed by selecting sequences with four or more cysteine residues from the Antimicrobial Peptides Database (APD) [35]. This set was manually curated, keeping only the sequences annotated at least with activities against bacteria, fungi or virus. In addition, incomplete sequences were removed. PS was composed of 385 sequences with size ranging from 16 to 90 amino acid residues. The negative data set (NS) was composed of a subset of Protein Data Bank (PDB), while in our previous work it was composed of random proteins predicted as transmembrane [20]. Initially, the protein sequences retrieved from the search by the term “NOT antimicrobial” were selected and then the sequences ranging from 16 to 90 residues were chosen. Therefore, redundant sequences were removed with a cutoff of 40% through CDHIT [36], with 1749 sequences remaining; from these, 385 were randomly selected to compose the NS. The blind data set (BS1) was composed of 75 sequences (approximately 20%) randomly selected from each set, PS and NS, totaling 150 sequences, while the training data set (TS) was composed of the remaining sequences, totaling 620 sequences (310 from each set). Similar negative data sets were used by Thomas et al. [23], Torrent et al. [24] and Fernandes et al. [25].

**Figure 3:**
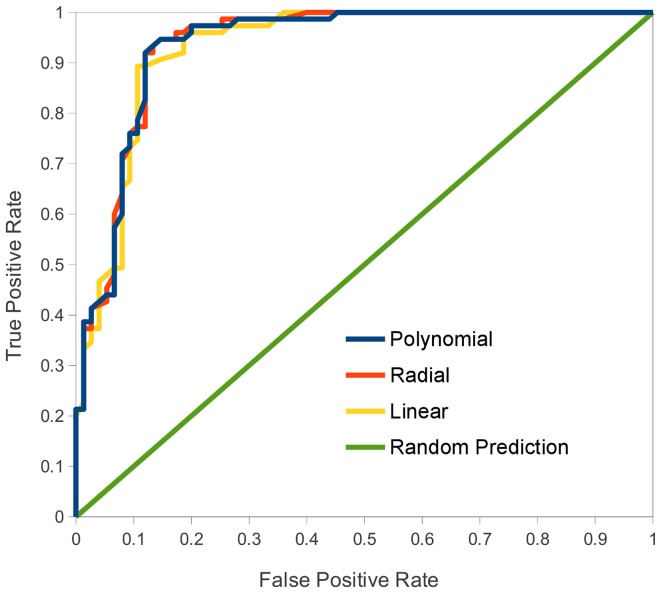
ROC curves for the CS-AMPPred models against the blind data set (BS1).

### Sequence Descriptors and Statistical Analysis

Preliminarily, nine structural/physicochemical properties were chosen: (i) average charge, (ii) average hydrophobicity, (iii) hydrophobic moment, (iv) amphipathicity, (v) α-helix propensity, (vi) flexibility and indexes of (vii) α-helix, (viii) β-sheet and (ix) loop formation. From our previous work [20], only three properties were considered (average hydrophobicity, hydrophobic moment and amphipathicity), being the average charge chosen instead the total charge. The secondary structure indexes were calculated as the average of weighted amino acid frequencies of Levitt (1977) [37]; flexibility was calculated as the average of amino acid flexibility, through the scale form Bhaskaran & Ponnuswamy (1988) [38]; the α-helix propensity was measured as the average energy to be applied in each amino acid for α-helix formation [39]; the amphipathicity was calculated as the ratio between hydrophobic and charged residues [3]; average hydrophobicity and hydrophobic moment were calculated using Eisenberg's scale [40]; the hydrophobic moment was given by Eisenberg's equation [40]; and the average charge was calculated as the net charge at physiological pH normalized by the number of residues. The final ensemble of sequence descriptors was defined through a principal component analysis (PCA). The nine descriptors were measured for the positive data set, and then the PCA was applied, subsequently the descriptors with redundant behavior or with little influence on variance were removed. Therefore, a two sided Wilcoxon-Mann-Whitney non-parametric test was applied for verifying the differences between the sequence descriptors in the PS and NS sets, with a critical value of 0.05. The statistical analyses were done through the R package for statistical computing (http://www.r-project.org).

**Table 1:** Evaluation of CS-AMPPred models against the individual cysteine-stabilized AMP classes and also PDB sequences which were not used in the data sets.

Model	α-defensins§	β-defensins§	CSαβ defensins§	Cyclotides§	Undefined§	PDB#
Linear	93.33	96.83	81.36	70.34	84.13	80.65
Polynomial	97.78	95.24	77.12	81.36	79.37	82.55
Radial	97.78	96.83	77.12	83.05	80.95	81.89

§ Antimicrobial Peptide Classes, values computed through equation 1 (Sensitivity).

# Non Antimicrobial Peptides, values computed through equation 2 (Specificity), using the 1364 sequences from PDB which were not included in NS.

### Support Vector Machine's Training and Validation

Three SVM models were developed through SVM Light [41], using the linear, polynomial and radial kernels. The training was done using the training set. An overview of the model's accuracy was estimated through a 5-fold cross validation, taking into account only the training data set. Therefore, the models were challenged against the blind data set, where the following parameters were measured:
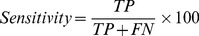
(1)

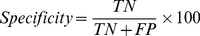
(2)

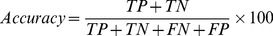
(3)

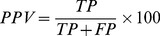
(4)

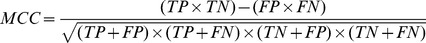
(5)Where TP is the number of true positives; FN, the false negatives; TN, the true negatives; FP, the false positives, PPV, the probability of positive prediction; and MCC, Matthews Correlation Coefficient.

**Table 2:** Benchmarking of prediction methods using the BS1.

Model	Sensitivity	Specificity	Accuracy	PPV	MCC	Reference
CS-AMPPred Linear	89.33	89.33	89.33	89.33	0.79	This work
CS-AMPPred Polynomial	94.67	85.33	90.00	86.59	0.80	This work
CS-AMPPred Radial	94.67	85.33	90.00	86.59	0.80	This work
ANFIS	94.67	76.00	85.33	79.78	0.72	[25]
CAMP SVM	93.33	78.67	86.00	81.40	0.73	[23]
CAMP Discriminant Analysis	98.67	70.67	84.67	77.08	0.72	[23]
CAMP Random Forest	90.67	**61.33**	76.00	70.10	**0.54**	[23]
SVM	84.00	**26.66**	**55.33**	**53.39**	**0.13**	[20]

**Table 3:** Benchmarking of prediction methods using the BS2.

Model	Sensitivity	Specificity	Accuracy	PPV	MCC	Reference
CS-AMPPred Linear	**69.81**	92.45	81.13	90.24	**0.64**	This work
CS-AMPPred Polynomial	77.36	90.57	83.97	89.13	**0.69**	This work
CS-AMPPred Radial	79.25	90.57	84.91	89.37	0.70	This work
ANFIS	100.00	100.00	100.00	100.00	1.00	[25]
CAMP SVM	88.68	96.23	92.45	95.92	0.85	[23]
CAMP Discriminant Analysis	90.57	98.11	94.34	97.96	0.89	[23]
CAMP Random Forest	96.23	**0.00**	**48.11**	**49.04**	**−0.14**	[23]
SVM	98.11	**67.92**	83.02	75.36	**0.69**	[20]

**Table 4:** Benchmarking of prediction methods using the BS1 and BS2.

Model	Sensitivity	Specificity	Accuracy	PPV	MCC	Reference
CS-AMPPred Linear	81.25	90.62	85.94	89.65	0.72	This work
CS-AMPPred Polynomial	87.50	87.50	87.50	87.50	0.75	This work
CS-AMPPred Radial	88.28	87.50	87.89	87.60	0.76	This work
ANFIS	96.88	85.94	91.41	87.32	0.83	[25]
CAMP SVM	91.41	85.94	88.67	86.67	0.77	[23]
CAMP Discriminant Analysis	95.31	82.03	88.67	84.14	0.78	[23]
CAMP Random Forest	92.97	**35.94**	**64.45**	**59.20**	**0.35**	[23]
SVM	89.84	**43.75**	**66.79**	**61.50**	**0.38**	[20]

Additionally, the sensitivity of each SVM model was tested separately against each peptide class: α-defensins, β-defensins, CSαβ defensins, cyclotides, hepcidins, hevein-like peptides, knottins, panaedins, tachplesins, θ-defensins, thionins and undefined. The group of undefined peptides encompasses peptides without a defined class and classes with fewer than five members. Furthermore, the 1364 sequences from PDB that were not included in NS were used for verifying the specificity of models.

### Benchmarking

The blind data set was used to compare the models generated in this study with the algorithms SVM, Discriminant Analysis (DA), and Random Forest (RF) from the Collection of Antimicrobial Peptides (CAMP) [23], an artificial neuro fuzzy inference system (ANFIS) [25] and also the SVM model generated by our previous work [20]. The assessment of each model was done through the parameters described in equations 1 to 5. Additionally, the blind data set from our previous work (BS2) [20] was also used as a second benchmarking assessment. BS2 is composed of 53 antimicrobial sequences with six cysteine residues extracted from APD and 53 proteins randomly generated predicted as transmembrane proteins [20]. There is an overlapping between the positive BS1 and BS2 sequences, once they were extracted from APD. Nevertheless there is no overlapping between the negative sequences, once in BS1 they were extracted from PDB. Furthermore the sequences from BS2 were randomly generated clearly showing any coinciding. A third assessment was done with the weighted average of the two benchmarks. BS1 and BS2 are available as Data Sets S1 and S2, respectively, in fasta format.

## Results and Discussion

The cysteine patterns are widely spread in several classes of biologically active peptides. These patterns are highly conserved and are responsible for keeping stable the structural folding. For this reason they are used for peptide classification [4], [20], [27]. Due to their multifunctionality, they have an enormous biotechnology potential [1], [2], [31], [32]. However, due to their multifunctional character, the identification of a single function without *in vitro* and/or *in vivo* tests is a very difficult task. As an example, we can cite the cyclotide parigidin-br1. This peptide was identified in leaves of *Palicurea rigida* [8] but was unable to control bacterial development, despite sharing 75% of identity with a bactericidal cyclotide named circulin b [42].

Among the possible activities, the antimicrobial one is a good target for prediction, since there are several databases dedicated to peptides with this kind of activity, such as APD [35] and CAMP [23]. Several models of antimicrobial activity prediction have been proposed by using such databases [20]–[25]. On the other hand, there are no non-antimicrobial peptide databases, which becomes an enormous challenge for constructing reliable models [20], [21], [25]. Several approaches have been proposed to overcome this problem, including the use of proteins with the annotation of non-antimicrobial from SwissProt or PDB [21], [23]–[25] or even using sequences predicted to have signal peptides or transmembrane portions [20], [25]. In this work, a subset of PDB was used as a negative data set, since the proteins in PDB are overall more curated than in other databases. The construction of the NS was done in three steps. First, the proteins from PDB were selected by searching for the term “NOT Antimicrobial”; second, the redundant sequences were removed with a cutoff of 40% of identity, ensuring that the non-redundant sequences represent a large sample space; and the last step was randomly selecting 385 sequences to compose the NS, avoiding an imbalance between NS and PS. In the case of CS-AMPPred, a NS composed of non-antimicrobial peptides with a similar number of cysteine residues would be ideal for validating it. However, there is no warranty that a peptide has no antimicrobial activity, unless it had been already screened against several microorganisms. In the case of parigidin-br1, it does not show bactericidal activity, but it was not tested as fungicidal [8].

Another problem involved in antimicrobial activity prediction is the size variation of the sequences. In this study, the sequences in PS can vary from 16 to 90 amino acid residues. To solve this problem two strategies have been proposed, (i) the use of a fixed length of amino acids [21] and (ii) the use of physicochemical properties as sequence descriptors [20], [23], [24]. Here, nine structural/physicochemical properties were chosen as sequence descriptors and then reduced to five descriptors by means of PCA (Figure 1). The final descriptors were average hydrophobicity, average charge, flexibility, and indexes of α-helix and loop formation (Figures 1b and 2). In addition, a two-sided Wilcoxon-Mann-Whitney non-parametric test was applied to verify statistical differences between PS and NS (Figure 2). The test indicates that there are differences between the sets. Similar results were observed by Torrent et al. [24]. These descriptors were chosen according to properties commonly related to AMPs, such as hydrophobicity and charge [20], [23], [25]. However, some descriptors can have the same behavior of others or even be expressionless, as observed for the hydrophobic moment (Figure 1). Therefore the PCA was done in order to select the descriptors strongly related to cysteine-stabilized antimicrobial peptides.

It is important to highlight that the use of net charge as a descriptor shows a clear bias. The charge can indefinitely increase or decrease with the sequence, while the other descriptors have a maximum and a minimum value. For this reason, in this study the average net charge at physiological pH was utilized. However, the use of averaged descriptors causes a second bias, since shuffled sequences will have the same averaged values [20], [43]. In our previous work the hydrophobic moment was proposed to solve this bias [20]. Nevertheless, the PCA shows that hydrophobic moment may not be a good property for the antimicrobial activity prediction of cysteine-stabilized peptides. Therefore, the properties must be carefully used together with the cysteine patterns of cysteine-stabilized AMPs. We state that this predictor must be used for cysteine stabilized peptides with a known pattern or a previously identified domain, since those descriptors are going to be only significant if the sequence is in its correct order.

In fact, the descriptors selection through PCA was useful for developing a more accurate antimicrobial activity prediction system, since the three kernel functions reach higher accuracies in the k-fold cross validation in comparison to our previous work [20]. While in this work the kernels reach accuracies of at least 84.19% (linear and radial kernels), in our previous work, the best accuracy on k-fold cross validation was 77% (polynomial kernel) [20]. Here, the best accuracy was also reached by the polynomial kernel, with 85.81%. This accuracy improvement indicates that the five selected descriptors (average hydrophobicity, average charge, flexibility, and indexes of α-helix and loop formation) showed higher efficiency than the four descriptors previously described by Porto et al. [20] (net charge at physiological pH, average hydrophobicity, hydrophobic moment and amphipathicity).

The receiver-operating characteristic (ROC) curves obtained for each kernel function against the blind data set (Figure 3) show that the models are underestimated in 5-fold cross validation, which also was observed in our previous work [20]. The accuracy of each model increases by ∼5% against the blind data set; the highest accuracies are obtained with the polynomial and radial kernels (90%), while the linear kernel shows 89.33% of accuracy. Furthermore, the MCC indicate that the tree models have a good quality prediction, with values of 0.79, 0.80 and 0.80 for linear, radial and polynomial kernels, respectively. In addition, the models have a PPV of 89.33%, 86.59% and 86.59%, respectively.

Although the model based on the polynomial kernel was the best one for overall prediction concerning the blind data set and 5-fold cross validation, the models based on linear and radial kernels were better predictors than the polynomial kernel for some individual classes, such as β-defensins, CSαβ defensins, cyclotides and peptides without a defined class (Table 1). However, the three CS-AMPPred models reach accuracies of 100% for the other classes (hepcidins, hevein-like peptides, knottins, panaedins, tachplesins, θ-defensins and thionins). However, the model based on polynomial kernel has a better prediction for non-antimicrobial peptides. By using the 1364 sequences from PDB which were not included in NS, the three models reach a specificity of ∼82% (Table 1). Despite this decoy, this value continues being considered as a good prediction.

The benchmarking with the BS1 indicates that the CS-AMPPred models have the best performances when compared to other systems; even the linear model, which was the worst CS-AMPPred model, was better than the other described algorithms (Table 2). However, using the BS2, the CS-AMPPred models were not as efficient as two CAMP algorithms (SVM and DA) and the ANFIS network (Table 3). This CS-AMPPred performance reduction with the BS2 was expected, since it contains antimicrobial sequences that belong only to three classes: α-defensins, CSαβ defensins and cyclotides. In these classes, the sensitivity of CS-AMPPred models is reduced when compared to the overall sensitivity from each model (Table 1). This reduction has an influence on the third benchmarking (Table 4), where the parameters of CS-AMPPred models, ANFIS network and CAMP's SVM and DA were more balanced.

In summary, the CS-AMPPred models obtained the best evaluations in a wider blind data set (Table 1). The CS-AMPPred models have the highest accuracies when tested on the general blind set and have a smaller number of input descriptors when compared with the CAMP models, which need 68 descriptors, once more showing the reliability of our principal component analysis. The CS-AMPPred models also achieve similar accuracies to other systems with more sequence descriptors, such as the artificial neural network (ANN) from Torrent et al. [24], which achieves an accuracy of 89.2% using eight descriptors; and the quantitative structure active relationship (QSAR) based ANN from Fjell et al. [22], which achieves an accuracy of 86.5% using 44 descriptors. However, the comparison with these two other systems must be made carefully since different data sets were used for assessment.

However, the most intriguing results were obtained with two other models, the SVM of our previous study [20] and the RF algorithm from CAMP [23], since they have a bad assessment, with MCC values below 0.7 (Tables 2, 3 and 4). The RF model did not have high specificity values for prediction of random protein sequences predicted as transmembrane (Table 3), and the SVM from our previous work did not have a good specificity for proteins from PDB (Table 2). These bad assessments show that when these prediction models are challenged with an unknown data set, their assessment parameters may not be the same. Indeed, a benchmarking event such as CASP for protein structure prediction is needed for comparing different algorithms and evaluates their performances in an actual blind data set.

In conclusion, this report presents the CS-AMPPred, an antimicrobial peptide predictor based on SVM Light [41]. The CS-AMPPred achieves predictions with enhanced reliability, showing an accuracy of 90% (polynomial model). Furthermore, it has a better assessment than previous systems in the overall blind data set. This better assessment is due to the specific target from our system, which was done aiming to predict antimicrobial activity for cysteine-stabilized peptides. In fact, this predictor can be used to predict the antimicrobial activity of several peptide sequences, since they have a regular cysteine pattern. The CS-AMPPred can be helpful for revealing the antimicrobial activity from multifunctional peptides. In addition, it can be useful for a prediction prior to synthesis of some predicted proteins in protein databases. In the future, sequences without antimicrobial activity will be predicted and tested *in vitro*.

### Availability and Requirements

A standalone version of CS-AMPPred was developed under the GNU/GPL 3.0 license and it is available for download at <http://sourceforge.net/projects/csamppred/>. The software was developed using the programming language PERL and compiled using the PERL Archiving Toolkit. CS-AMPPred runs on any Linux machine and its download is free for academic use; commercial users should contact the authors for license.

## Supporting Information

Data Set S1 The blind data set 1 (BS1) in fasta format. It was composed of 75 sequences randomly selected from each set (PS and NS) totaling 150 sequences.
(FAS)

Data Set S2 The blind data set 2 (BS2) in fasta format. BS2 is composed of 53 antimicrobial sequences with six cysteine residues extracted from APD and 53 proteins randomly generated predicted as transmembrane proteins [20].
(FAS)
